# Abdominal obesity-related risk factors in children from public schools of Barbacena, Minas Gerais, Brazil

**DOI:** 10.1590/1984-0462/2022/40/2020354

**Published:** 2021-10-04

**Authors:** Mariana das Dores Paiva Canuto, Adriele Vidal Lucas Silva, João Victor Martins, Marlene de Melo Fonseca, Nathália Sernizon Guimarães, Anne Danieli Nascimento Soares, Júnia Maria Geraldo Gomes

**Affiliations:** a Instituto Federal de Educação, Ciência e Tecnologia do Sudeste de Minas Gerais, Campus Barbacena, Barbacena, MG, Brazil.; b Universidade Federal de Ouro Preto, Ouro Preto, MG, Brazil.

**Keywords:** Child, Obesity, abdominal, Pediatric obesity, Food consumption, Anthropometry, Criança, Obesidade abdominal, Obesidade pediátrica, Consumo de alimentos, Antropometria

## Abstract

**Objective:**

To evaluate the sociodemographic and lifestyle factors associated with abdominal obesity in children from public schools in Barbacena, state of Minas Gerais, Brazil.

**Methods:**

This is a cross-sectional study conducted on 326 students aged 7 to 9 years from public schools in the urban area of the city. Anthropometric data included body weight, height, body mass index according to age, waist circumference, and waist-to-height ratio. Food consumption was evaluated using the Previous Day Food Questionnaire. Abdominal obesity was assessed based on waist circumference and waist-to-height ratio. The bivariate and multivariate analyses were performed by logistic regression, estimating the crude and adjusted odds ratio (OR), with 95% confidence interval.

**Results:**

The prevalence of overweight was 30.7%; whereas the prevalence of abdominal obesity was 9.2 and 12.6% according to waist circumference and waist-to-height ratio, respectively. Boys (OR 2.76; 95%CI 1.22–6.25) and children from central schools (OR 2.73; 95%CI 1.08–6.80) presented an increased chance of abdominal obesity according to waist circumference. Abdominal obesity according to waist-to-height ratio was associated with the central location of the schools (OR 2.18; 95%CI 1.02–4.63) and the habit of skipping supper (OR 2.01; 95%CI 1.00–4.09).

**Conclusions:**

The findings showed that being a boy, studying in a central school, and skipping supper were the main risk factors associated with abdominal obesity.

## RESUMO

**Objetivo:**

Avaliar os fatores sociodemográficos e de estilo de vida associados à obesidade abdominal em crianças de escolas públicas de Barbacena, Minas Gerais.

**Métodos:**

Trata-se de estudo transversal realizado com 326 escolares de sete a nove anos, matriculados em escolas da zona urbana da cidade. As variáveis antropométricas avaliadas foram peso, estatura, índice de massa corpórea por idade, circunferência da cintura e relação cintura-estatura. O consumo alimentar foi registrado pelo Questionário Alimentar do Dia Anterior. A obesidade abdominal foi avaliada com base na circunferência da cintura e relação cintura-estatura. As análises bivariada e multivariada foram realizadas por meio de regressão logística, estimando-se a *Odds Ratio* (OR) bruta e ajustada, com os intervalos de confiança de 95% (IC95%).

**Resultados:**

A prevalência de excesso de peso foi de 30,7%, enquanto a prevalência de obesidade abdominal foi de 9,2 e 12,6%, de acordo com circunferência da cintura e relação cintura-estatura, respectivamente. Crianças do sexo masculino (OR 2,76; IC95% 1,22–6,25) e de escolas centrais (OR 2,73; IC95% 1,08–6,80) tiveram maior chance de apresentar obesidade abdominal segundo a circunferência da cintura. A obesidade abdominal, de acordo com a relação cintura-estatura, associou-se à localização central das escolas (OR 2,18; IC95% 1,02–4,63) e à omissão da ceia (OR 2,01; IC95% 1,00–4,09).

**Conclusões:**

Os achados mostraram que ser do sexo masculino, estudar em escola central e omitir a ceia foram os principais fatores de risco associados à obesidade abdominal.

## INTRODUCTION

Obesity is considered a serious public health issue, especially in children.^1^ Children and adolescents are more vulnerable to present nutritional disorders due to the intense process of growth and development, which results in increased energy and nutritional demands.^1^ Childhood obesity is associated with several comorbidities, such as dyslipidemia, diabetes mellitus, and hypertension, which can persist until adulthood.^2^

Anthropometry is widely used in clinical practice because it is a low-invasive method, of low cost, and of good reproducibility.^1^ The body mass index (BMI) is the main instrument used to diagnose individuals’ obesity. Although it does not provide an accurate estimate of body composition, this index has a good correlation with body fat and, mainly, with risk of mortality. Conversely, waist circumference (WC) and waist-to-height ratio (WtHR) are better indicators for estimating abdominal obesity and can be used as predictors of cardiovascular disease (CVD) risk.^3^ Therefore, the combined use of these three anthropometric indices (BMI, WC, and WtHR) allows more reliable estimates of the evaluation of abdominal obesity and, consequently, of the risk of developing CVD among children.^1^

Differences between the prevalence of overweight among students in public and private schools is well-studied;^4^ ^,^ ^5^ however, studies that assess the nutritional status of schoolchildren in the same school system, differentiating them by the geographic location of schools in the urban area of the same city, are still scarce.^6^ There are also few studies that analyzed factors associated with the development of abdominal obesity among children, considering that most research focuses on investigating only the presence of general obesity.^2^ ^,^ ^4^ ^,^ ^6^ Among abdominal obesity-related risk factors in children, studies show genetic and environmental characteristics such as physical inactivity, socioeconomic level, inadequate eating habits, and duration and intensity of sleep.^1^ ^,^ ^2^ ^,^ ^7^ Understanding the environmental factors related to abdominal obesity is important for identifying risk groups and enabling the implementation of food and nutrition education initiatives especially aimed at this population segment, aiming at the prevention and treatment of this clinical condition.

In this context, the objective of this study was to evaluate the sociodemographic and lifestyle factors (diet and physical activity – PA) associated with abdominal obesity in children from state public school system in the city of Barbacena, state of Minas Gerais, Brazil.

## METHOD

**Figure 1:**
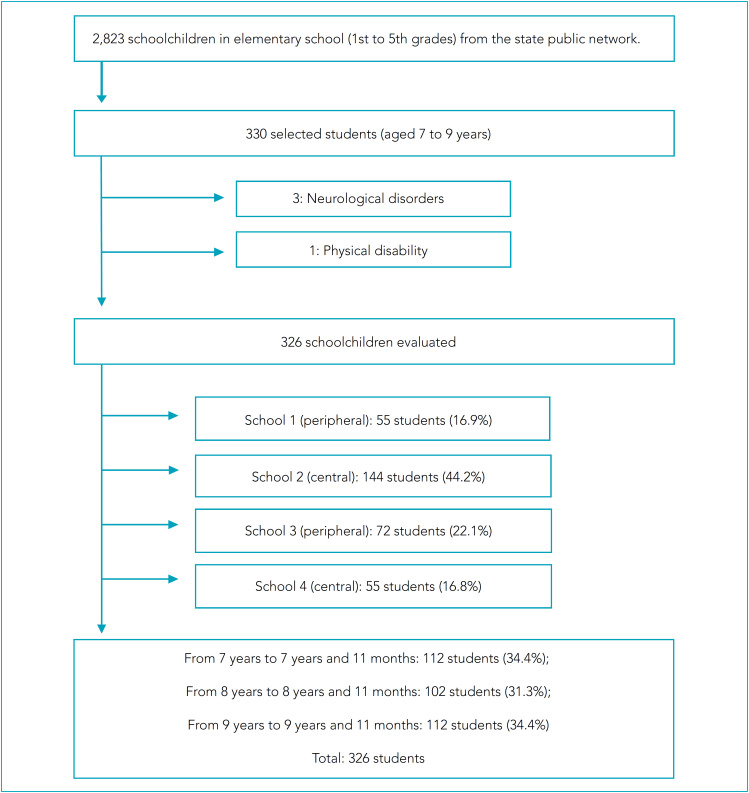
Flowchart of selection of the study population. Barbacena, Minas Gerais, Brazil, 2020.

This is a cross-sectional study conducted on students, aged 7–9 years, enrolled in the public school system in the urban area of Barbacena, Minas Gerais. Data collection took place between July 2019 and February 2020. Due to differences in the cutoff points used in nutritional assessment, children with special needs, such as Down Syndrome or neurological disorders, were excluded as well as children who had physical disabilities (Figure 1).

According to the State Superintendence of Education of Barbacena, based on the school census of the year 2018, in the entire urban area the total number of students from the 1^st^ to the 5^th^ grades of elementary education in the state public network was equal to 2,823 students, divided into 12 schools. Those aged between seven and nine years were selected because they are aware of their school stage. First, a cluster sampling was carried out, and four schools were chosen by random drawing in each region of the city. Next, there was a simple random sampling, in which the selection of students per school and grades (from the 1^st^ to the 5^th^ grades) was carried out by simple random drawing until the required number of students was completed. Sample size was calculated using a 95% confidence interval (95%CI), a 5% sampling error, and a 37% prevalence of overweight in schoolchildren,^8^ corresponding to the minimum sample of 318 schoolchildren. Schools that participated in the study were divided into central (2) and peripheral (2), according to their geographical location (Figure 1).

For the anthropometric evaluation, techniques proposed by Lohman et al.^9^ were used. For weight measurement, a calibrated Balmak Slimbasic® portable scale was used, with a maximum capacity of 150 kg and subdivision of 100 g. For the assessment of height, a 2-m Cescorf® inelastic measuring tape, 1-mm subdivision, was used, fixed on a straight, smooth wall and without baseboard. BMI was calculated using the formula weight (kg)/height^2^ (m^2^). For the classification of nutritional status, the Z-score was adopted for the BMI according to age (BMI-for-age) and the height according to age (height-for-age) indices, considering the curves of the World Health Organization.^10^ According to BMI-for-age, students were divided into two groups: 1) no excess body weight, including schoolchildren classified as underweight and normal weight; 2) with excess body weight and obesity, including those classified as overweight, obese, and severely obese. WC was measured in the smallest circumference using a Cescorf® inelastic measuring tape, and the presence of abdominal obesity was classified according to Freedman et al.^11^ WtHR was estimated by dividing WC and height, and values of ≥0.5 were considered indicative of abdominal obesity.^12^

The Previous Day Food Questionnaire (PDFQ, version 3) was the dietary survey used in the study, structured into six meals (breakfast, mid-morning snack, lunch, mid-afternoon snack, dinner, and supper), with 21 food groups each.^13^ This is an illustrated instrument that obtains data on the food consumption of schoolchildren from the previous day.^13^ The application occurred according to the technique described in the manual of the Department of Nutrition of Universidade Federal de Santa Catarina.^14^

The food groups of the PDFQ were classified as protective foods (vegetables and greens, vegetable soup, fruits, and natural juices) and risk foods (chocolate milk, sweets and desserts, chips, fast-food meals, industrialized snacks, artificial fruit juices, and soft drinks).^5^ ^,^ ^15^ The consumption of protective foods was considered adequate when consumed more frequently or equal to six times a day; and inadequate when the listed foods were consumed less than five times a day. Conversely, the consumption of risk foods was classified as inadequate when its intake occurred more than three times a day; and as adequate when it occurred twice a day or less.^15^

The Previous Day Physical Activity and Food Questionnaire (PDPAFQ) was used to assess PA levels.^16^ This validated instrument provides a global score on the performance of PA through the sum of the scores concerning physical exercises performed and reported by children. It contains 11 types of activities that can be performed in three different intensities (slow, fast, and very fast). To classify the PA level, three different weights were assigned, according to the intensity with which the physical exercises were performed: weight one for lightly performed activities (slowly); weight three for moderately performed activities (fast); and weight nine for vigorously performed ones (very fast).^16^ The total score ranged from 0 to 99 points, and students were classified as little active (up to 33 points), moderately active (from 34 to 66 points), and extremely active (above 67 points).^17^

Data were tabulated in Microsoft Office Excel® and analyzed using the STATA® software, version 13.0. The Shapiro-Wilk test was used to verify the normality of continuous variables. Pearson's chi-square test, Fisher's exact test, and Yates’ correction chi-square test were used to assess categorical variables. Logistic regression was adopted to obtain the crude Odds Ratio (OR) and that adjusted for age in order to verify the association between overweight, abdominal obesity, and independent variables. All variables that obtained p <0.20 were included in the regression. The level of significance was set at 95% (p≤0.05).

All procedures involving human subjects were approved by the Human Research Ethics Committee of the Instituto Federal de Educação, Ciência e Tecnologia do Sudeste de Minas Gerais (opinion No. 3.588.082). To participate in the study, written informed assent was obtained from all subjects. Parents or guardians signed the informed consent in order to consolidate the minor's participation.

## RESULTS

A total of 326 schoolchildren participated in the study, most of them were girls (51.8%), with a balanced number of subjects between the age groups (33% each), enrolled in central schools (61%), and little active (98.8%) (Table 1). Most participants had breakfast (86.2%), lunch (94.2%), mid-afternoon snack (85%), and dinner (86.8%), but did not have mid-morning snack (79.8%) and supper (56.4%) (Table 1).

**Table 1:** Distribution of height-for-age and BMI-for-age indices according to sex, age, geographic location, consumed meals, physical activity, and consumption of protective and risk foods. Barbacena, Minas Gerais, Brazil, 2020.

			Total		Height-for-age			BMI-for-age		
			n	%	Adequate	Inadequate	p-value	No EBW	EBW	p-value
Sex										
	Girls		169	51.80	167	2	0.268	124	45	0.100
	Boys		157	48.20	157	0		102	55	
Age group										
	7 to <8 years old		112	34.50	110	2	0.624	81	31	0.467
	>8 to <9 years old		101	31.10	101	0		69	32	
	>9 to <10 years old		112	34.50	112	0		75	37	
Geographical location of the school										
	Central		199	61.00	199	0	0.151	133	66	0.222
	Peripheral		127	39.00	125	2		93	34	
Meals										
	Breakfast									
		No	45	13.80	45	0	0.743	30	15	0.677
	Mid-morning snack									
		No	260	79.80	258	2	0.636	180	80	0.942
	Lunch									
		No	19	5.80	19	0	0.887	16	3	0.147
	Mid-afternoon snack									
		No	49	15.00	48	1	0.278	33	16	0.745
	Dinner									
		No	43	13.20	43	0	0.753	31	12	0.673
	Supper									
		No	184	56.40	183	1	0.682	126	58	0.706
Physical activity										
	Little active		322	98.80	321	1	0.024	222	100	0.229
	Moderately active		4	1.20	3	1		4	0	
Protective foods										
	Adequate		14	4.30	14	0	0.916	10	4	0.562
	Inadequate		312	95.70	310	2		216	96	
Risk foods										
	Adequate		200	61.40	199	1	0.624	145	55	0.075
	Inadequate		126	38.60	125	1		81	45	

Height-for-age: height according to age; BMI-for-age: body mass index according to age; EBW: excess body weight and obesity. No excess body weight: children with underweight and normal weight; Excess body weight and obesity: overweight, obese, and severely obese children. Chi-square, Fisher's exact, and Yates’ correction chi-square tests were performed.

**Figure 2:**
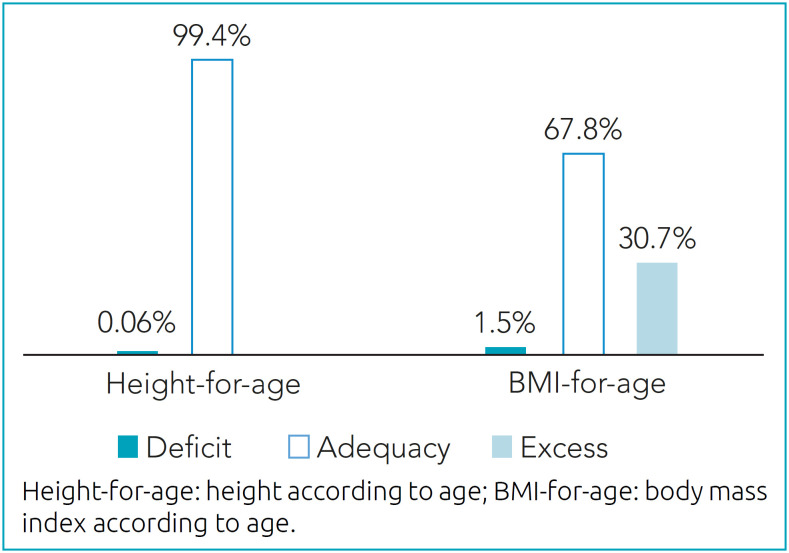
Nutritional status of schoolchildren aged seven to nine years (n=326), according to height-for-age and BMI-for-age indices. Barbacena, Minas Gerais, 2020.

Only 0.6% (n=2) of the students had low height-for-age. The prevalence of excess body weight and obesity was 30.7% (n=100), accounting for 16% (n=52) overweight, 10.4% (n=34) obesity, and 4.3% (n=14) severe obesity. Underweight was observed for 1.5% (n=5) of the participants (Figure 2).

The prevalence of abdominal obesity was 9.2% (n=30) and 12.6% (n=41), according to WC and WtHR, respectively (Table 2). Most schoolchildren with inadequacies related to excess body weight and obesity were boys, considering BMI-for-age (16.9%; n=55), WC (6.4%; n=21), and WtHR (7.4%; n=24) (Tables 1 and 2). The inadequacy of the height-for-age index was associated with performance of PA (p=0.024) (Table 1).

**Table 2:** Distribution of waist circumference and waist-to-height ratio according to sex, age, geographic location, consumed meals, physical activity, and consumption of protective and risk foods. Barbacena, Minas Gerais, Brazil, 2020.

			Abdominal obesity according to WC			Abdominal obesity according to WtHR		
			Absent	Present	p-value	Absent	Present	p-value
Sex								
	Girls		160	9	0.012	152	17	0.155
	Boys		136	21		133	24	
Age group								
	7 to <8 years old		98	14	0.257	99	13	0.773
	>8 to <9 years old		94	7	0.378	87	14	1.000
	>9 to <10 years old		103	9		98	14	
Geographical location of the school								
	Central		175	24	0.025	168	10	0.041
	Peripheral		121	6		117	31	
Meals consumed according to PDFQ-3								
	Breakfast							
		No	41	4	0.600	40	5	0.487
	Mid-morning snack							
		No	235	25	0.407	224	35	0.170
	Lunch							
		No	19	0	0.151	18	1	0.282
	Mid-afternoon snack							
		No	43	6	0.424	44	5	0.393
	Dinner							
		No	41	2	0.210	38	5	0.536
	Supper							
		No	164	20	0.236	155	29	0.034
Physical activity								
	Little active		292	30	0.678	281	41	0.583
	Moderately or extremely active		4	0		4	0	
Protective foods								
	Adequate (>6 times/day)		11	3	0.128	13	1	0.455
	Inadequate (<5 times/day)		285	27		272	40	
Risk foods								
	Adequate (<2 times/day)		179	21	0.307	172	28	0.329
	Inadequate (>3 times/day)		117	9		113	13	

WC: waist circumference; WtHR: waist-to-height ratio; PDFQ-3: Previous Day Food Questionnaire; height-for-age: height according to age; BMI-for-age: body mass index according to age. Chi-square, Fisher's exact, and Yates’ correction chi-square tests were performed.

More than a third of the sample (38.7%; n=126) consumed excess risk foods. However, no significant association was found between the variables of food consumption and the classification regarding anthropometric variables (Table 1).

Abdominal obesity according to WC was associated with boys (p=0.012) and the central location of schools (p=0.025). Abdominal obesity according to WtHR was associated with skipping supper (p=0.034) and the central location of schools (p=0.041) (Table 2).

Table 3 presents the analysis of crude and adjusted OR for the evaluation of abdominal obesity according to WC and WtHR. It was observed that being a boy (OR 2.76; 95%CI 1.22–6.25) and studying in the central region (OR 2.73; 95%CI 1.08–6.80) were risk factors for developing abdominal obesity according to WC, even after adjustments. According to WtHR, studying in the central region (OR 2.18; 95%CI 1.02–4.63) and skipping supper (OR 2.01; 95%CI 1.00–4.09) were risk factors for abdominal obesity.

**Table 3:** Association between abdominal obesity and independent variables in schoolchildren aged seven to nine years. Barbacena, Minas Gerais, Brazil, 2020.

		Abdominal obesity according to WC		Abdominal obesity according to WtHR	
		OR 95%CI	Adj OR 95%CI	OR 95%CI	Adj OR 95%CI
Sex					
	Girls	1	1	1	1
	Boys	2.74 (1.21–6.19)	2.76 (1.22–6.25)	1.61 (0.83–3.13)	1.60 (0.82–3.10)
Geographic location					
	Peripheral	1	1	1	1
	Central	2.76 (1.09–6.96)	2.73 (1.08–6.89)	2.15 (1.01–4.57)	2.18 (1.02–4.63)
Protective foods					
	Inadequate	1	1	–	–
	Adequate	0.34 (0.09–1.32)	0.36 (0.09–1.38)	–	–
Mid-morning snack consumption					
	Yes	–	–	1	1
	No	–	–	1.96 (0.73–5.21)	1.96 (0.74–5.26)
Supper consumption					
	Yes	–	–	1	1
	No	–	–	2.52 (1.01–4.13)	2.01 (1.00–4.09)

WC: waist circumference; WtHR: waist-to-height ratio; OR: Odds Ratio; Adj OR: Odds Ratio adjusted for age; 95%CI: 95% confidence interval. Logistic regression was performed to obtain the Odds Ratio values.

## DISCUSSION

As the main result of this study, being a boy, studying in public schools located in the central region, and skipping supper were considered risk factors for abdominal obesity in schoolchildren aged between seven and nine years. Corroborating the present results, a study carried out in the southern region of Brazil showed that being a boy was associated with high BMI and/or abdominal obesity in children.^5^ According to the literature, girls are more dissatisfied with their body image in relation to boys, a behavior that may reflect a greater concern with body weight gain. In addition, there is strong cultural and media pressure to adopt thinness as an ideal standard for women.^18^ Although research shows that boys tend to be more active than girls, those with excess body weight have a lower level of PA and more hours of sedentary behaviors.^19^

In this study, the central geographic location of schools was a risk factor for abdominal obesity in schoolchildren. A study carried out in the city of Sorocaba, state of São Paulo,^6^ on children from municipal public schools, found a higher prevalence of obesity among children enrolled in central schools compared with those who studied in peripheral schools. A possible explanation for the higher prevalence of abdominal obesity in the central schools of Barbacena, Minas Gerais, is that the environment around and within these schools may contain limitations for the performance of non-sedentary activities, for adequate diet and/or the choice of healthy habits, constituting an obesogenic environment.^20^ Lourenço et al.,^20^ when assessing the influence of the school environment on the nutritional status of preschoolers in the public school system of Macaé, Rio de Janeiro, observed that children older than five years of age from schools with a broad social support network in their surroundings had less chance of being overweight. On the other hand, the school with more cafeterias and food advertisements in the area had a higher prevalence of obesity.^20^ The school, as well as the family, has a great influence on the causality of childhood obesity and is a privileged place to promote healthy eating habits and PA.^20^ Public policies for the prevention and control of obesity can be formulated based on the assessment of nutritional status according to geographic location and, especially, considering the factors related to the obesogenic environment in schools.^20^

The habit of skipping supper was observed as a risk factor for the development of abdominal obesity in the present study. Skipping meals is considered a risk behavior, as such meals can be replaced with unhealthy snacks (mainly those made of processed and ultra-processed foods), which contribute to the increase in calorie intake and the consequent body weight gain.^21^ Skipping breakfast, for example, can impair school performance, satiety and weight control, and favor the desire to consume caloric snacks,^7^ but data demonstrating the consequences and risks associated with the habit of not having supper are still scarce in the literature. Silva et al.^21^ observed that supper was the most skipped meal by 708 children (66%) and adolescents (77.3%) aged between seven and 14 years old in Juiz de Fora, Minas Gerais, and that the lower frequency of meals was associated with increased BMI values. The authors suggested that the frequent consumption of meals is related to better weight control due to the increase in postprandial energy expenditure, with increased overall thermic effect of food in regular meal patterns. In addition, not skipping meals could contribute to keeping constant blood glucose and insulinemia throughout the day, promoting better control of appetite and satiety.^21^

A high prevalence of excess body weight and obesity (30.7%) was observed in this study, with a value similar to that found by the Consumer Expenditure Survey (*Pesquisa de Orçamentos Familiares* – POF) (33.5%)^22^ and also by the Food and Nutritional Surveillance System (*Sistema de Vigilância Alimentar e Nutricional* – SISVAN) (29.5%).^23^ The prevalence of overweight and obesity found in this study was higher than that observed in Campina Grande (state of Paraíba) (21.5%)^7^ and in eight municipalities in the state of Santa Catarina (Florianópolis, Joinville, Blumenau, Chapecó, Criciúma, Jaraguá do Sul, Lages, and Joaçaba) (20.9%);^4^ however, it was lower than that found in another study carried out in Itajaí, Santa Catarina (44%), for the same age group.^24^ Obesity, characterized by the excessive accumulation of body fat, poses risks to human health, particularly during childhood, due to the great chances that this condition will persist during adulthood and lead to the early emergence of complications such as type 2 diabetes, hypercholesterolemia, arterial hypertension, obstructive sleep apnea syndrome, musculoskeletal disorders and other diseases.^25^ The increase in the prevalence of childhood obesity and the consequent increase in the risk of developing CVD among the younger population has become a public health issue in Brazil and worldwide, reflecting the situation of nutritional transition.^4^ ^,^ ^24^

In this study, only 0.6% of the students had low height-for-age, this prevalence being lower than that found by POF (6.8%)^22^ and by a study carried out in Campina Grande (2.4%).^7^ Height deficits indicate a multicausal problem, which results from the interaction between dietary patterns and health problems in people with unfavorable socioeconomic conditions. It is worth mentioning that short stature may be associated with genetic patterns, which does not exclusively mean nutritional risk. Nevertheless, the expression of the genetic potential results from its interaction with environmental factors.^26^

Almost the entire sample of this study (98.8%) was classified as little active or sedentary, a higher frequency than that observed in other national studies included in a systematic review on the level of PA and nutritional status of Brazilian children, which found prevalence of physical inactivity from 22.6 to 93.5%.^19^ The high variability of the applied questionnaires, the use of non-validated instruments, and the lack of consensus on the cutoff points to define physical inactivity contribute to divergences in the results. Some studies characterize sedentary behavior as the time spent watching television, playing video games, using tablets, cell phones, and computers.^19^ ^,^ ^27^ However, the screen time only comprises part of the time of little activity, as it excludes other sedentary activities such as sitting at school and commuting, for example.^27^ The instrument used in the present research was validated for children and includes the modes of transport used to go to school and the PA performed during the day. The increase in sedentary lifestyle among children is worrisome, as it has positive associations with excess body weight.^27^ The regular performance of PA helps to decrease the levels of body adiposity and blood lipids, in addition to contributing to the increase in bone mineral density and the improvement of mental health.^16^

The low intake of fresh foods and minimally processed foods, as well as the high consumption of processed and ultra-processed foods, have been observed in this and other studies.^4^ ^,^ ^7^ ^,^ ^20^ ^,^ ^25^ The reduced consumption of vegetables, greens, and foods by children is alarming from a nutritional point of view, considering that these foods are essential for the quality of the diet and help to maintain a healthy body weight.^15^ ^,^ ^28^ In the long-term, the inadequate intake of these foods contributes to the emergence of nutritional deficiencies and to the development of chronic noncommunicable diseases such as arterial hypertension, diabetes, dyslipidemia, obesity, and neoplasms.^28^ Therefore, it is important to encourage the adoption of healthy eating habits since childhood, as this is a behavior that favors the promotion and maintenance of health in adulthood^29^ and assists in the prevention and control of excess body fat.^29^

Among the limitations of this study, the cross-sectional design can be mentioned, which makes it difficult to establish causal relationships between the evaluated variables. Another limitation was the lack of assessment of income and school environment to characterize the population, considering that these variables can influence the development of diseases and health problems, resulting in changes in nutritional status.^30^ The cognitive maturity of the age group under study must also be taken into account, as many children have difficulties in communicating, compromising the quality of the provided information. Furthermore, it is worth emphasizing as a limitation the use of PDFQ-3 for the assessment of food intake, as this instrument does not provide data on the exact amount of consumed food and was applied in just one day, which does not reflect the students’ usual food consumption. Nevertheless, it is noteworthy that all the applied questionnaires were validated for the age group evaluated and the applied food survey considers that children have not yet reached the stage of abstract reasoning and, therefore, have reduced capacity to report information about frequencies. and quantities.^13^

The authors conclude that, among all the evaluated variables, being a boy, being enrolled in a central school, and skipping supper increased the chances of anthropometric inadequacies related to abdominal obesity. This finding helps to guide public policies and nutritional intervention actions for groups with higher cardiovascular risk, indicated by the accumulation of abdominal fat.
